# Supplementary material for: Adverse pregnancy outcomes among women in Norway with gestational diabetes using three diagnostic criteria
Source: PLoS One. 2023 Jul 6;18(7):e0280750. doi: 10.1371/journal.pone.0280750 (PMC10325062; doi:10.1371/journal.pone.0280750)
Supplement: Attachment — Submitted filename: Response to Reviewers.docx [file pone.0280750.s003.docx]

Point-by-point response to reviewers

**Reviewer 1:**

**Comment**: I would recommend the authors to make two clarifications:
The authors pooled 4 studies: 2 cohorts and 2 RCTs and have chosen to make a pooled analysis.
One sentence in the method section:
“Studies that only included specific subgroups (e.g. obese women only) or without the core data were not considered for inclusion” suggests that the number of studies to start with was chosen according to selection criteria. This could be clarified.

***Reply****:* Thank you for your overall positive review and your valuable comments. We agree that we can provide clarity to this part. We have now added to this paragraph the following sentence: *“The four studies were merged to perform a pooled analysis.” (page 5)*

We agree that this sentence may be misinterpreted. We have now changed the sentence to *“Only studies that had core data and were not based on specific selection criteria (e.g. obese women only) were considered for inclusion.”(p.5)*

**Comment**: Can the authors justify why they have chosen to make a pooled analysis (adjusted for study cohorts) instead of making a meta-analysis. Although this second approach has less statistical power it may help observing study specific effects. Please justify and may be discuss in the strength and limitation section.

***Reply****: Thank you for suggesting an alternative statistical analysis. In principal we have performed statistical analysis comparable with an IPD-meta analysis. However, the analysis was not performed in relation with a systematic review, as the studies included were chosen based on a consortium of studies with glucose data. As statistical power was limited also in the pooled analysis, we did not consider presenting results for these relatively rare outcomes from each individual study (as often done as a first step in a meta-analyses). We did however use the same principles as in a meta-analysis for harmonization of variables etc, and a simpler search did not identify other Norwegian studies, expect one study of obese pregnant women. Nevertheless, as we had not strictly followed all methodology recommended for an IPDMA we chose to call this a "pooled analysis".*

**Comment**: Although it is well explained in the main text, the sentence in the abstract: Asians had a lower risk of delivering large-for-gestational-age infants than Europeans […] can be misleading to readers with limited knowledge in birth weight categorisation. Although you informed (’but maternal glucose values were similarly positively associated with birthweight in all ethnic groups’) I think that there is still a risk of miss-interpretation.

***Reply****: This sentence could arguably be clearer, and we have now adjusted it as follows below. We hope that it will be acceptable that the abstract now consists of 301 words.*

*“Asians had a lower risk of delivering large-for-gestational-age infants than Europeans, when applying national birthweight references, but maternal glucose values were similarly positively associated with birthweight in all ethnic groups".*

**Comment:** In theory, as Asian babies are clearly off-chart when it comes to LGA and SGA categorization (based on the Norwegian scales) the analyses cannot be performed as they are meaningless. I would suggest to remove Asian from the LGA analyses.

**Reply**: *Thank you for your suggestion. We agree that not having an* *ethnicity-specific definition for LGA is a limitation, and this is also discussed in the paper. Unfortunately, there are no customized charts for this group in Norway. In our considered opinion, it is better to employ the national references, which are regularly used in clinical practice for all pregnant women living in Norway, than to completely remove the Asian group from the analysis. As LGA based on a Norwegian reference population is not a very precise measure for Asian women, we have additionally performed linear regression using birthweight z-score (table 4) to explore the association with maternal glucose.*

**Comment**: The discussion is very clear and concise. However, all the comma separators have been substituted by dots. This should be corrected of course.

**Reply***: I am sorry for this error that appeared at submission. This have now been corrected*.

**Reviewer 2:**

**Comment**: In Figure 1 what are 'STORK Groruddalen', 'STORK Rikshospitalet', 'Fitfor Delivery', & 'TRIP' - all these acronyms must be explained in the figure.

***Reply****: Thank you for your valuable comments and suggestions. These are actually not acronyms but the original study titles (Groruddalen and Rikshospitalet are places/hospitals in Norway). TRIP is an acronym for Training in pregnancy. We have added an explanation to the figure text to make this clearer.*

***“****Study names listed in the top boxes. TRIP: Training in pregnancy.” (p. 7)*

**Comment**: The standard nomenclature for International Association of Diabetes and Pregnancy Study Groups (IADPSG) and World Health Organization 2013 (WHO 2013) recommendations is IADPSG or WHO 2013.
Using 2013(superscript)WHO criteria is not standard way of describing. Please show why are these criteria depicted this way - 2013WHO and not WHO 2013 criteria.

***Reply****: We chose superscript for the years (2013, 2017 and 1999) to increase readability and make it easier to follow for the reader, as these criteria are mentioned a large number of times and it can be confusing with all the numbers.*

**Comment**: The study must make it clear they are not using the full 3 point WHO 2013 or IADPSG but instead using a modified 2 point IADPSG or modified WHO 2013 criteria without 1 hour. The 1 hour time point is an essential part of the IADPSG criteria and will independently adds another one third of cases. This must be stated clearly a limitation that only fasting and 2 hour timepoints are used and thus they are not reflecting the IADPSG full criteria but only comparing the 2 time points criteria within the WHO 2013 criteria.

***Reply****: We agree that this is an important point that should be emphasized. It has been mentioned as a limitation in the discussion, but to provide even more clarity, we have added the points suggested by you to this paragraph.*

*“Finally, we used a modified ^2013^WHO criteria with only two timepoints, as data for 1-hour glucose concentrations were not collected in our study. The prevalence of GDM by the ^2013^WHO criteria would presumably have been higher with the addition of the 1-hour timepoint.” (p. 22)*

**Comment**: It was shown that Asians had a lower risk of delivering large-for-gestational-age babies. This is not helpful as Asians are smaller size and their babies size norms may be lower. Are customised charts used for Asians, e.g. in relation to mother height and weight and ethnicity?

***Reply****: See also response to reviewer 1. LGA was calculated using the same Norwegian reference population as used in clinical practice, for classification of LGA/SGA by sex and gestational age. Ethnically customized centile charts are not available in Norway (being a small country), and using customized charts from other countries could also potentially introduce bias, as mean birthweight in Norway is generally higher than for example UK (both for “White” and several immigrant groups). As we agree that LGA based on a Norwegian reference population is not a very precise measure for Asian women, we have instead performed linear regression using birthweight z-score (table 4) to explore the association with maternal glucose. We believe this analysis provides more useful knowledge for the non-European groups.*

**Comment**: However can these findings be applied to Asia in terms of the timepoints? e.g. fasting and relation to baby birthweights etc. Please refer to an Asian paper - Sri Lanka paper with criteria similar to Norwegian for fasting time point, where a similar aspect of changes in the criteria affect the number of cases
- Dias T, Siraj SHM, Aris IM, Li LJ, Tan KH. Comparing Different Diagnostic Guidelines for Gestational Diabetes Mellitus in Relation to Birthweight in Sri Lankan Women. Front Endocrinol (Lausanne). 2018 Nov 15;9:682. doi: 10.3389/fendo.2018.00682. PMID: 30524375; PMCID: PMC6262349.

***Reply****: thank for you suggesting this relevant paper. We have added this reference in the discussion, as it provides valuable insight into the subject discussed.*

*“A study by Dias et al. also supports our findings, showing that ^2013^WHO criteria is associated with greater birthweight in Sri Lankan pregnant women. (p.21)*
