# Supplementary material for: Adverse pregnancy outcomes among women in Norway with gestational diabetes using three diagnostic criteria
Source: PLoS One. 2023 Jul 6;18(7):e0280750. doi: 10.1371/journal.pone.0280750 (PMC10325062; doi:10.1371/journal.pone.0280750)
Supplement: Attachment — Submitted filename: Point-by-point response 08.8.22.docx [file pone.0280750.s004.docx]

Point-by-point response to reviewers

Response to editor:

**Comment: Suggest to use the abbreviation LGA also in the abstract.**

Reply: I have now added the abbreviation LGA to the abstract and use this instead.

**Comment:**  **Abstract / Results: In case of a lower or upper limit of 1.0 in a 95% CI, please add as many digits as necessary to indicate whether the 95% CI contains the 1 or not. Mentioning p-values in the main text will then become unnecessary.**

Reply: As suggested, I have now removed the p-values from the main text and added extra digits to the 95 CI% with a lower or upper limit of 1.0.

**Comment: Is it correct that those mothers identified by the 2013WHO and 2017Norwegian criteria but not diagnosed and treated by 1999WHO criteria were mothers with an FPG between 5.1 and 6.9 mmol/L, irrespectively of 2HG? If so, the authors might consider to mention this definition throughout the manuscript to describe this group instead of the lengthy wording used in the manuscript (after justifying it based on the different GDM definitions).**

Reply: This is not entirely correct. Mothers identified by the 2013-WHO and 2017-Norwegian criteria but not diagnosed and treated by 1999-WHO criteria were mothers with an FPG between 5.1 (or 5.3) and 6.9 mmol/L *and* with a 2H glucose < 7.8 mmol/L. To keep this as clear as possible to the readers we believe it’s necessary to refer to it in this way, although we agree it’s a bit lengthy.

**Comment: "Distributions of all potential covariates were tested for normality." Which test was used? Were all continuous covariates assumed to follow a normal distribution based on the test results? Please add this information to the main text.**

Reply: The information about the tests is now added to the main text. *“Assumptions for statistical analysis were tested and distributions of all potential covariates were checked for normality using Tests of Normality and by inspection of probability plots, which confirmed that these variables followed a normal distribution.” (p.7-8)*

**Comment: Table 1: Please clarify that % values refer to column %.**

Reply: To clarify this we have now changed the legend for Table 1 to:

*Table 1. Characteristics and pregnancy outcomes in total study sample and according to their glucose tolerance status, using three diagnostic criteria for gestational diabetes. For each column data are presented as mean ± SD or n (%).*

**Comment: Results: ORs are sometimes given with 2, sometimes with 3 digits. This should be handled in a uniform manner, apart from 1.0 in 95% CIs as mentioned above.**

Reply: Thank you for pointing this out. We have corrected this, and all OR’s are now given with 3 digits.

**Comment: It is unclear to me why the sensitivity analysis was done and what it adds. Please explain this in some detail.**

Reply: In the majority of studies similar to ours, researchers exclude participants that have received an intervention/treatment. As much as we would like to do the same, we were unable to due to limited number of participants in our studies. Excluding all participants diagnosed and treated based on the 1999-WHO criteria would leave us with reduced power to explore the outlined associations. The sensitivity analysis was therefore mainly performed to verify the results achieved by the regression analyses where adjustments for treatment and a known diagnosis were made to the model as an alternative to excluding women. To further clarify this point, the following part has been added to the manuscript:

“As a sensitivity analysis, and to verify the results achieved by the analyses where adjustment for treatment and a known diagnosis were made to the model, we repeated the same analysis after excluding participants who were diagnosed and treated based on the ^1999^WHO criteria as an alternative to excluding women.” (p.8-9).

**Comment:**
**- Table 4:
Were the conditions for applying a linear regression between maternal glucose and offspring's birthweight checked? In particular, is it plausible to assume a linear relationship instead of a J-shaped or U-shaped one?**

Reply: The conditions for a linear regression were checked, and the relationship between maternal glucose (fasting and 2HG) and birthweight z-score was linear. The sentence: *“The conditions for a linear regression were checked, confirming a linear relationship between maternal glucose values and offspring birthweight”* has been added to the methods section (p. 9).

*** Presumably, FPG and 2HG were not mutually adjusted due to collinearity. This should be explained in the text.**

Reply: It is correct that FPG and 2HG were not mutually adjusted and this is mentioned in the table text. However, it can be explained in the text as well. One sentence about this has now been added under Statistical Analyses: *“We did not mutually adjust for FPG and 2HG due to collinearity.” (p.9)*

*** Asian, unadjusted: "P" should read "95% CI"**

Thank you for this remark. It has now been corrected.

*** The sign "-" should not be used as a separator between the lower and upper limit of a 95% CI, as it is already needed to indicate negative values. Suggest to use "," or ";" instead (consistently throughout the manuscript)**

We have now changed this sign into to the suggested “,” throughout the manuscript.

**- In the spirit of Open and Reproducible Science, the analysis code (or SPSS syntax) should be made available in an online repository together with a data dictionary, and the respective URL should be mentioned in the Methods section. Can the data also be made available, and if not, why not?**

Reply: The reason for not being able to share data has been explained thoroughly upon submission under “Data Availability”. This is copy of our statement previously sent:

*The datasets generated and/or analyzed during the current study are not publicly available due to the dataset containing potentially sensitive data. The editors can access data (in de-identified form) used in the manuscript, code book, and analytical code upon request. The project manager will contribute to the access being provided under appropriate conditions. However, research data for this publication include identifying health information subject to confidentiality. It is therefore not possible to share raw data publicly.*

Reviewer #2:

**Comment: Using a prefix, superscript year before the criteria is highly unconventional in medical literature for diabetes (usually reserved for atomic particles); and may also lead to formatting, spacing and search issues when published. Would still suggest in order of preference, for the 'year' , to be after the criteria and non superscript with space or dash or bracket if needed; or to be after the criteria as postfix.**

Reply: Thank you for your suggestion. We have changed this throughout the manuscript, and the criteria are now listed as: WHO-1999, WHO-2013 and Norwegian-2017.
