# Supplementary material for: Adverse pregnancy outcomes among women in Norway with gestational diabetes using three diagnostic criteria
Source: PLoS One. 2023 Jul 6;18(7):e0280750. doi: 10.1371/journal.pone.0280750 (PMC10325062; doi:10.1371/journal.pone.0280750)
Supplement: Attachment — Submitted filename: PLOS one Adverse outcomes GDM Norway.docx [file pone.0280750.s005.docx]

PLOS one: Adverse pregnancy outcomes among women in Norway with gestational

diabetes using three diagnostic criteria

The authors present the results of a cohort study that aimed to compare perinatal outcomes between women diagnosed and treated for GDM by the more stringent WHO 1999 diagnostic criteria and women who during that period would have theoretically been diagnosed as having GDM by the lower threshold 2013 and 2017 criteria but were hence not labelled as GDM and thus untreated. This subject has been previously assessed in other studies in different countries and with varying combinations of GDM diagnostic thresholds. The cohort was somewhat unusually constructed by pooling data from 2 cohort studies and two RCTs which might insert an unmeasurable selection bias and influence generalizability. I am not sure whether this a revision of a previously submitted manuscript, but I have not had the opportunity to review it previously.

Overall, this a well thought out paper that is providing some additional information to the many papers that have been published attempting to retrospectively analyze the perinatal impact of changing GDM diagnostic criteria.

Below are my itemized individual comments and questions:

1. Abstract, results: The term “women diagnosed with GDM by **all** three criteria” might be confusing. Do you mean that women that would have been considered positive by either of the three diagnostic criteria i.e all women with either FPG>5.0 or 2hr >7.7 which would infer pooling of the GDM women in the cohorts or is the intent to say that women who were diagnosed in either of the groups (non-pooling) had increased rates of LGA. I assume the latter as this is more in line with what is stated in the results.
2. Introduction 1^st^ paragraph: I would reconsider stating that “Gestational diabetes mellitus (GDM) is associated with … foetal and neonatal mortality”. While there is good data to support the association with other perinatal outcomes, this is not true for stillbirth and neonatal death. The reference provided (1) does not support this statement.
3. Methods: Justify the grouping of south and east Asian ethnicity under “Asian”. Do these two distinct ethnic backgrounds have similar BMI/insulin resistance levels and rate of GDM related outcomes? Many studies have suggested that South Asians are a distinct group with regards to glucose handling.
4. Methods: I would like to understand why fetal deaths that occurred after glucose testing were excluded and not reported as an adverse outcome? This potentially removes some of the most severe cases of poor glycemic control.
5. I am trying to understand how many women diagnosed with GDM by WHO -99 criteria failed lifestyle modification. This is not reported in Table 1 but in the methods it is mentioned that only 12 needed “such pharmacological treatment” which I will assume means either insulin or oral hypoglycemics (correct me if I am wrong). If this is the case, then the rate of lifestyle modification failure was a bit less than 3.8%. This is much less than the reported rate in the literature (20- 40%) and is even more surprising in light of the fact that the FPG threshold for diagnosis and treatment was > 6.9 mmol/L which would have led to a high probability of treatment failure. When examining the mean FPG in the WHO-99 GDM group, the mean FPG was 5.0+/- 0.6 thus it seems that there were very few women with significantly elevated FPG raising questions regarding how representative this cohort is of the GDM population in general ( see comment my comment in the preamble). Perhaps add categorical data on the FPG and 2 hr values (i.e FPG 5.1-5.5; 5.6-6.0; 6.0-6.4; 65-6.9) for clarification of the population characteristics.
6. Results: It is stated that “Women diagnosed with GDM by any criteria had a higher rate …”. I do not see a group defined as meeting ANY of the criteria thus this result is not visible in any of the tables and not indicated in the planned analysis (similar to comment 1 above). Please ensure that these group comparisons confirm to the planned analysis and to the groupings presented in the tables.
7. Figures: pay attention to formatting issues. Figure 1 – not sure is needed in the main paper – could be in supplementary data. I would prefer that figure 1 be a diagram of the different cohorts by diagnostic criteria and the number that would have been identified and treated in each.
8. Table 2: Not clear what the first column represents. Add heading. I would also make it clear in the title (and text) that there is a global risk reported for each outcome followed by a stratified analysis by BMI and ethnicity. In the footer define what BMI define Normal, overweight and obesity. Also – in the footer it says that the variables adjusted for are age. gestational weeks at delivery, parity and study cohort while in the text it states that there was also adjustment for treatment by the WHO-1999 criteria. Please clarify and ensure uniformity in reporting.
9. In both Table 2 and 3: I am not sure why under the outcome (LGA, CS etc) there is a subcategory of the diagnostic criteria. This is confusing and unnecessary as this is self-evident from the column identification. Also, Table 3 formatting is unusual with different Tables for each outcome and separate footnotes. Should align with format of Table 2.
10. Also, in the title of Table 3 (and in text) it would be good to spell out for the reader that this analysis is for the subgroup of those that would have GDM by the newer criteria but were UNDIAGNOSED AS GDM AND THUS UNTREATED.
11. As I was re-reading, another possibility is to add to Table 2 the untreated aOR with a footnote explaining the different cohort structure. This would allow the reader to compare visually the untreated vs the combination of treated and untreated cohorts. With appropriate Table design this could be not too complex.
12. If needed – Table 4 could easily be a supplementary table.
13. Discussion: Is the statement “Those retrospectively identified by the Norwegian-2017 and WHO-2013 criteria, but who were not diagnosed and treated by the WHO-1999 criteria (i.e., women with moderately elevated fasting glucose only) also had an increased risk of cesarean section and operative vaginal delivery after adjustment for confounders.” Accurate? In Table 3, that shows the results for “untreated GDM”, ONLY LGA was significant. Clarify and adjust discussion as needed.
14. At the end of the first paragraph in the discussion you state that “moderately elevated fasting glucose, when unidentified and untreated, is associated with several adverse outcomes” and that the increased risk was “…primarily based on elevated 2HG values.”. I do not see any separate analysis showing the effect of the individual abnormal 75 gram OGTT value except for the effect on z score birthweight which is definitely not an “adverse outcome” in itself. New results should not be entered into the discussion. Please clarify and if needed adjust manuscript accordingly.
15. When comparing the results to what is known in the literature you cite studies from Canada. For your interest one of the first studies from Canada is Am J Obstet Gynecol 2015;212:224.e1-9.
16. The last sentence in the conclusion states that “What remains unanswered and can be established by randomized trials is whether treating mild hyperglycaemia benefits women and their offspring, leading to an improvement in perinatal outcomes” is a bit bombastic and does not take into consideration ACHOIS (to a lesser degree) and the NICHD study (N Engl J Med 2009; 361:1339-1348).I would consider changing this.
