# Supplementary material for: Adverse pregnancy outcomes among women in Norway with gestational diabetes using three diagnostic criteria
Source: PLoS One. 2023 Jul 6;18(7):e0280750. doi: 10.1371/journal.pone.0280750 (PMC10325062; doi:10.1371/journal.pone.0280750)
Supplement: Attachment — Submitted filename: Response_to_Reviewers_17.11.22.docx [file pone.0280750.s006.docx]

Point-by-point response to reviewers

**Reviewer 3**

1. Abstract, results: The term “women diagnosed with GDM by **all** three criteria” might be confusing. Do you mean that women that would have been considered positive by either of the three diagnostic criteria i.e all women with either FPG>5.0 or 2hr >7.7 which would infer pooling of the GDM women in the cohorts or is the intent to say that women who were diagnosed in either of the groups (non-pooling) had increased rates of LGA. I assume the latter as this is more in line with what is stated in the results.

**Respond**: Thank you for your thorough review and overall positive comments.

We agree that the wording can be confusing, and as you suggest, we have edited the statement to:

*“Compared to the non-GDM group, women diagnosed with GDM by either of the three criteria had an increased risk of large-for-gestational-age infants (adjusted odds ratios (OR) 1.7-2.2).” (p.2)*

2. Introduction 1^st^ paragraph: I would reconsider stating that “Gestational diabetes mellitus (GDM) is associated with … foetal and neonatal mortality”. While there is good data to support the association with other perinatal outcomes, this is not true for stillbirth and neonatal death. The reference provided (1) does not support this statement.

**Respond**: Foetal and neonatal mortality is now removed from the sentence, and the reference is updated to ensure that the content corresponds with the phrase. The new sentence is:

*“Gestational diabetes mellitus (GDM) is associated with increased risk of macrosomia, caesarean section, preeclampsia and preterm delivery. (p.4)*

3. Methods: Justify the grouping of south and east Asian ethnicity under “Asian”. Do these two distinct ethnic backgrounds have similar BMI/insulin resistance levels and rate of GDM related outcomes? Many studies have suggested that South Asians are a distinct group with regards to glucose handling.

**Respond**: This is a good point. However, out of the 223 Asian women included in this study, only 39 had East Asian origin. We did not have statistical power to study the relationship between glucose levels and the related outcomes in these two groups separately, but sensitivity analyses (not shown) did not suggest that they differed. We therefore made the decision to combine them. The mean BMI and mean birthweight were quite similar in the two groups, but, not surprisingly, South Asians had a somewhat higher prevalence of GDM (42% vs 24% in East Asians, using 2013-WHO criteria). This is already added as a limitation in the discussion.

“However, nearly all non-European women came from one study and the majority of Asians were of South Asian origin.” (p.25)

4. Methods: I would like to understand why fetal deaths that occurred after glucose testing were excluded and not reported as an adverse outcome? This potentially removes some of the most severe cases of poor glycemic control.

**Respond**: Fetal deaths in this study include abortion<22 weeks, stillbirth≥22 weeks and neonatal death. The majority of these occurred before the first OGTT, and we lack outcome data (birthweight, birth complications, sex etc.) from most of these. After excluding those lacking data on birthweight, we were left with only one fetal death (with normal glucose values) which was then excluded. We have changed the order of the words in the sentence to make it clearer that infants with missing birthweight were excluded first, and then fetal deaths (and figure 1 shows that only one fetal death was excluded).

*“After excluding women with multiple pregnancies, those lacking glucose values, infants with missing birthweight and foetal deaths, the study sample consisted of 2970 mother-child pairs.”*

5. I am trying to understand how many women diagnosed with GDM by WHO -99 criteria failed lifestyle modification. This is not reported in Table 1 but in the methods it is mentioned that only 12 needed “such pharmacological treatment” which I will assume means either insulin or oral hypoglycemics (correct me if I am wrong). If this is the case, then the rate of lifestyle modification failure was a bit less than 3.8%. This is much less than the reported rate in the literature (20- 40%) and is even more surprising in light of the fact that the FPG threshold for diagnosis and treatment was > 6.9 mmol/L which would have led to a high probability of treatment failure. When examining the mean FPG in the WHO-99 GDM group, the mean FPG was 5.0+/- 0.6 thus it seems that there were very few women with significantly elevated FPG raising questions regarding how representative this cohort is of the GDM population in general ( see comment my comment in the preamble). Perhaps add categorical data on the FPG and 2 hr values (i.e FPG 5.1-5.5; 5.6-6.0; 6.0-6.4; 65-6.9) for clarification of the population characteristics.

**Respond**: Your understanding is correct regarding how many women failed lifestyle modification - only 12 women needed insulin or metformin, based on available information in the hospital records. Several points may influence this rather low number. Some women may have started with lifestyle modification but later needed medication, in which case their medication may have been overlooked and their treatment registered incorrectly. Furthermore, universal screening was performed in our four cohorts as opposed to risk-factor based screening often carried out in many comparable studies. With that, we include women with a milder form of GDM, and our study may be more representative of real-life low risk populations. We have also touched upon in the discussion that our study included women with less overweight/obesity than the general population which may also have a certain impact.

6. Results: It is stated that “Women diagnosed with GDM by any criteria had a higher rate …”. I do not see a group defined as meeting ANY of the criteria thus this result is not visible in any of the tables and not indicated in the planned analysis (similar to comment 1 above). Please ensure that these group comparisons confirm to the planned analysis and to the groupings presented in the tables.

**Respond**: The term “diagnosed with GDM by any criteria..” means *any of the three* criteria. To clarify this further, we have changed the sentence to:

*“All three groups of women diagnosed with GDM (according to WHO-1999, WHO-2013 and Norwegian-2017 criteria respectively) had a higher rate of LGA neonates compared to their non-GDM counterparts, while higher rates of macrosomia (birthweight>4000g) were only found in those diagnosed by Norwegian-2017 and WHO-2013 criteria.”(p.9)*

7. Figures: pay attention to formatting issues. Figure 1 – not sure is needed in the main paper – could be in supplementary data. I would prefer that figure 1 be a diagram of the different cohorts by diagnostic criteria and the number that would have been identified and treated in each.

**Respond**: We accept your suggestion of moving Figure 1 to supplementary data. As we mainly look at these four cohorts as one pooled dataset, our main focus is not showing GDM rates in each cohort etc. However, the data on number of women identified and treated in each cohort was published in the first paper from this consortium, and a reference to this specific paper for those interested in more information is already added, and can be found under number 12:

*“Detailed study methods for the pooled data set have been previously described (12)..” (p.5)*

8. Table 2: Not clear what the first column represents. Add heading. I would also make it clear in the title (and text) that there is a global risk reported for each outcome followed by a stratified analysis by BMI and ethnicity. In the footer define what BMI define Normal, overweight and obesity. Also – in the footer it says that the variables adjusted for are age. gestational weeks at delivery, parity and study cohort while in the text it states that there was also adjustment for treatment by the WHO-1999 criteria. Please clarify and ensure uniformity in reporting.

**Respond**: Thank you for pointing out that this table was difficult to interpret; we appreciate the opportunity to clarify. The first column shows crude (unadjusted) analyses, while the next columns show adjusted analysis for each criteria (not a stratified analysis by BMI and ethnicity). This is now clearly presented in the table. The definition of BMI categories is now added to the footer.

Further, the analyses are also adjusted for treatment by WHO1999, as stated in the text and shown in the table, as well as pre-pregnancy BMI and ethnicity. The footnote stated that the analysis was “Additionally adjusted for age, maternal smoking, parity and study cohort” – these are the variables we adjusted for but which are not shown separately in the table. The actual number for the rest (treatment, BMI etc) are listed in the tables.

In order to avoid any confusion, we have amended the footer to explicitly define the adjusted analysis. The first applies to the column regarding WHO 1999 criteria, while the second applies to the WHO-2013 and Norwegian-2017 criteria.

** Adjusted for BMI group and ethnicity, as shown. Additionally adjusted for age, gestational weeks at delivery, parity and study cohort.*

**^∞^***Adjusted for GDM diagnosed by WHO-1990 criteria, BMI group and ethnicity, as shown. Additionally adjusted for age, gestational weeks at delivery, parity and study cohort.*

New title for table 2: *Crude and adjusted analyses of risk of large-for-gestational-age, total cesarean section and operative vaginal delivery, by GDM-criteria.* (p.13).

9. In both Table 2 and 3: I am not sure why under the outcome (LGA, CS etc) there is a subcategory of the diagnostic criteria. This is confusing and unnecessary as this is self-evident from the column identification. Also, Table 3 formatting is unusual with different Tables for each outcome and separate footnotes. Should align with format of Table 2.

**Respond**: Table 2: the subcategory of diagnostic criteria is listed mainly to show the crude analyses for each criteria, and the unadjusted risk would be impractical to present otherwise. In table 3 we agree that we could have just presented one row and typed “GDM” instead of GDM and the respective criteria. We have also merged the three tables into one and placed the footnotes in the end of the table to align with the format of table 2.

10. Also, in the title of Table 3 (and in text) it would be good to spell out for the reader that this analysis is for the subgroup of those that would have GDM by the newer criteria but were UNDIAGNOSED AS GDM AND THUS UNTREATED.

**Respond**: Thank you for this suggestion. In this sensitivity analysis, we repeat the same analysis as in table 2, but **after** excluding women who were diagnosed and treated according to the WHO-1999 criteria. One could of course turn it around and give it a title as suggested. However, what we wish to highlight here is that this is the exact same analysis as table 2 (in which we adjusted for the treatment effect) but it is performed after excluding diagnosed/treated women. The analysis presented in Table 3 was performed mainly for statistical reassurance, to support and verify our main analysis (Table 2).

11. As I was re-reading, another possibility is to add to Table 2 the untreated aOR with a footnote explaining the different cohort structure. This would allow the reader to compare visually the untreated vs the combination of treated and untreated cohorts. With appropriate Table design this could be not too complex.

**Respond**: Thank you for your suggestion. This could be possible, but, as this table is already complex with a lot of data and details, we would prefer to keep it as it is.

12. If needed – Table 4 could easily be a supplementary table.

**Respond**: We would like to include this table to demonstrate the important point that the use of LGA as an outcome representing excessive fetal growth is challenging, as the definition of LGA is not ethnicity-specific. Thus, very few ethnic Asian babies were born LGA. With the help of the analyses presented in this table, we show that the effect of elevated glucose on birthweight was similar across ethnic groups.

13. Discussion: Is the statement “Those retrospectively identified by the Norwegian-2017 and WHO-2013 criteria, but who were not diagnosed and treated by the WHO-1999 criteria (i.e., women with moderately elevated fasting glucose only) also had an increased risk of cesarean section and operative vaginal delivery after adjustment for confounders.” Accurate? In Table 3, that shows the results for “untreated GDM”, ONLY LGA was significant. Clarify and adjust discussion as needed.

**Respond**: Our main analyses are presented in table 2 and our goal was to include all women without having to exclude a large number of participants as this might lead to power limitations and less representativity. However, with advice from study statistician and suggestions from reviewers in the first revision round, we included a sensitivity analysis (table 3) to support and verify the results of our main analysis. This is explained under the heading “statistical analysis”:

*“As a sensitivity analysis, and to verify the results achieved by the analyses where adjustment for treatment and a known diagnosis were made to the model, we repeated the same analysis after excluding participants who were diagnosed and treated based on the WHO-1999 criteria.” (p.8)*

This sensitivity analyses gave almost identical effect estimates as the main analyses (table 2), although, when women with GDM by WHO-1999 were excluded, statistical significance was only reached for LGA. This was probably due to the decreased population size in this analysis, which is why our conclusion is based on the analyses in table 2 to a greater extent.

14. At the end of the first paragraph in the discussion you state that “moderately elevated fasting glucose, when unidentified and untreated, is associated with several adverse outcomes” and that the increased risk was “…primarily based on elevated 2HG values.”. I do not see any separate analysis showing the effect of the individual abnormal 75 gram OGTT value except for the effect on z score birthweight which is definitely not an “adverse outcome” in itself. New results should not be entered into the discussion. Please clarify and if needed adjust manuscript accordingly.

**Respond**:
We regret that our wording has been unclear. Our intention was not to enter new results in this part of the paper, but rather to underline that the WHO-1999 criteria are primarily based on elevated 2HG values. To avoid misunderstandings, we have added to the sentence the words “which are”.

*“Taken together, these findings indicate that moderately elevated fasting glucose, when unidentified and untreated, is associated with several adverse outcomes that were not observed in women where GDM was detected and treated according to WHO-1999 diagnostic criteria, which are primarily based on elevated 2HG values. (p.19)*

15. When comparing the results to what is known in the literature you cite studies from Canada. For your interest one of the first studies from Canada is Am J Obstet Gynecol 2015;212:224.e1-9.

**Respond**: Thank you for making us aware of this relevant paper. We have now added this study along with the other studies from Canada in the reference list.

16. The last sentence in the conclusion states that “What remains unanswered and can be established by randomized trials is whether treating mild hyperglycaemia benefits women and their offspring, leading to an improvement in perinatal outcomes” is a bit bombastic and does not take into consideration ACHOIS (to a lesser degree) and the NICHD study (N Engl J Med 2009; 361:1339-1348).I would consider changing this.

**Respond**: You are correct that if read alone, this sentence doesn’t take into consideration the studies of Crowther and Landon. However, this sentence is connected to the previous sentences and must be read in light of them. Our data demonstrates that women with moderately elevated fasting glucose levels have an increased risk of adverse pregnancy outcomes, but we need more data, and especially RCT’s, demonstrating whether treating women falling into this particular group (moderately elevated fasting glucose but a low 2HG value) has clinical value. Although both studies (ACHOIS and NICHD) demonstrated a benefit of treatment of GDM, both of these studies employed criteria focused on elevated post-load glucose values (ACHOIS used criteria similar to WHO 1999, and NICHD included only women with fasting glucose <5,3 mmol/l combined with elevated 1-, 2- or 3- hour values) thereby demonstrating the need for more information on the effect of treating women with moderately elevated fasting glucose values only.

We have now modified this sentence to make it more nuanced:

*“What remains unanswered and can be established by randomized trials is whether treating mild fasting hyperglycaemia benefits women and their offspring, leading to an improvement in perinatal outcomes.”*

**Reviewer 4:**

Reviewer #4: The main query is understanding the groups in the WHO 2013 and Norwegian 2017 cohorts that were offered treatment. In Table 1 e.g the WHO 2013 column for the non-GDM has 119 and for GDM 199. From the text it is stated that they were offered treatment if they met the WHO 1999 criteria . Presumably this meant that their 2-hr levels were between 7.8 and 8.5 mmol/l for those that were "non-GDM" and ≥8.5 for the "GDM" group? If this is a correct interpretation it would help to have this spelt out more clearly. The dilemma with labelling them as WHO 2013 GDMs is as you have noted that it misses the group who would have been diagnosed by the 1-hr value of ≥10.0.
My recommendation is that including this group does not add significantly to the paper and could be removed.

Table 1Normal weight should be ≤ 24.9 not ≥24.9

Minor correction the HAPO study being published in the USA is hyperglycemia, not hyperglycaemia.

**Respond**: Thank you for your comments and suggestions.

It is correct that women in the study were offered treatment if they met the WHO 1999 criteria – that is, fasting glucose ≥7.0 mmol/l and/or 2-hour glucose (2HG) ≥7.8 mmol/l. In the analyses we have adjusted for the treatment effect (or excluded treated women in the sensitivity analyses, table 3).

Women with GDM by WHO-2013 criteria, after controlling for or excluding treatment by WHO-1999 criteria, are those with fasting glucose 5.1-6.9 mmol/l and 2-hour glucose 2HG <7.8 mmol/l.

Women with GDM by Norwegian-2017 criteria, after controlling for or excluding treatment by WHO 1999-criteria, are those with fasting glucose 5.3-6.9 mmol/l and 2-hour glucose <7.8 mmol/l.

We have tried to explain this in the paper, both under statistical analyses and under results, see page 8 and 13:

*“Doing so allowed us to identify the group of women with an elevated fasting blood glucose only (fasting glucose 5.1-6.9 mmol/l and 2HG <7.8 mmol/l for WHO-2013, and fasting glucose 5.3-6.9 mmol/l and 2HG <7.8 mmol/l for Norwegian-2017 criteria) who were untreated.”*

*“After adjusting for confounders and for treatment by the WHO-1999 criteria (thereby expressing the risk related to having fasting glucose 5.1-6.9 mmol/l while 2HG <7.8 mmol/l) the OR for LGA for this group was 1.70 (95% CI 1.2,2.5,) compared to non-GDM women (both fasting glucose <5.1 mmol/l and 2HG <7.8 mmol/l).(p.13)” etc*

The reason we didn’t exclude treated women completely from our analyses is that it would affect representativity and result in low power to detect associations with GDM. Therefore, we have instead adjusted for the treatment effect when studying this group.

It is unclear if you suggest removing the women diagnosed by WHO 2013 criteria, as we do not have information on women with elevated 1-hour glucose values. We maintain that it is important to include this group, despite our acknowledged limitation, as it allows us to compare the outcomes associated with two different thresholds for fasting glucose: 5.1 mmol/l for WHO 2013 and 5.3 mmol/l for Norwegian 2017. Of these, the WHO 2013 criteria are most widely used, and therefore relevant for comparison.

The symbols have now been corrected as per your suggestion, so is the spelling of “hyperglycemia”.
